# Using Machine Learning Imputed Outcomes to Assess Drug-Dependent Risk of Self-Harm in Patients with Bipolar Disorder: A Comparative Effectiveness Study

**DOI:** 10.2196/24522

**Published:** 2021-04-21

**Authors:** Anastasiya Nestsiarovich, Praveen Kumar, Nicolas Raymond Lauve, Nathaniel G Hurwitz, Aurélien J Mazurie, Daniel C Cannon, Yiliang Zhu, Stuart James Nelson, Annette S Crisanti, Berit Kerner, Mauricio Tohen, Douglas J Perkins, Christophe Gerard Lambert

**Affiliations:** 1 Center for Global Health Department of Internal Medicine University of New Mexico Health Sciences Center Albuquerque, NM United States; 2 Department of Computer Science The University of New Mexico Albuquerque, NM United States; 3 New Mexico Behavioral Health Institute Las Vegas, NM United States; 4 TwoFoldChange Consulting Bozeman, MT United States; 5 Iterative Consulting Albuquerque, NM United States; 6 Division of Epidemiology, Biostatistics, and Preventive Medicine Department of Internal Medicine The University of New Mexico Health Sciences Center Albuquerque, NM United States; 7 Biomedical Informatics Center George Washington University Washington, DC, DC United States; 8 Department of Psychiatry & Behavioral Sciences University of New Mexico Health Sciences Center Albuquerque, NM United States; 9 Semel Institute for Neuroscience and Human Behavior David Geffen School of Medicine University of California Los Angeles Los Angeles, CA United States; 10 Division of Translational Informatics Department of Internal Medicine University of New Mexico Health Sciences Center Albuquerque, NM United States

**Keywords:** bipolar, mood, mania, depression, pharmacotherapy, self-harm, suicide, machine learning, psychotherapy

## Abstract

**Background:**

Incomplete suicidality coding in administrative claims data is a known obstacle for observational studies. With most of the negative outcomes missing from the data, it is challenging to assess the evidence on treatment strategies for the prevention of self-harm in bipolar disorder (BD), including pharmacotherapy and psychotherapy. There are conflicting data from studies on the drug-dependent risk of self-harm, and there is major uncertainty regarding the preventive effect of monotherapy and drug combinations.

**Objective:**

The aim of this study was to compare all commonly used BD pharmacotherapies, as well as psychotherapy for the risk of self-harm, in a large population of commercially insured individuals, using self-harm imputation to overcome the known limitations of this outcome being underrecorded within US electronic health care records.

**Methods:**

The IBM MarketScan administrative claims database was used to compare self-harm risk in patients with BD following 65 drug regimens and drug-free periods. Probable but uncoded self-harm events were imputed via machine learning, with different probability thresholds examined in a sensitivity analysis. Comparators included lithium, mood-stabilizing anticonvulsants (MSAs), second-generation antipsychotics (SGAs), first-generation antipsychotics (FGAs), and five classes of antidepressants. Cox regression models with time-varying covariates were built for individual treatment regimens and for any pharmacotherapy with or without psychosocial interventions (“psychotherapy”).

**Results:**

Among 529,359 patients, 1.66% (n=8813 events) had imputed and/or coded self-harm following the exposure of interest. A higher self-harm risk was observed during adolescence. After multiple testing adjustment (*P*≤.012), the following six regimens had higher risk of self-harm than lithium: tri/tetracyclic antidepressants + SGA, FGA + MSA, FGA, serotonin-norepinephrine reuptake inhibitor (SNRI) + SGA, lithium + MSA, and lithium + SGA (hazard ratios [HRs] 1.44-2.29), and the following nine had lower risk: lamotrigine, valproate, risperidone, aripiprazole, SNRI, selective serotonin reuptake inhibitor (SSRI), “no drug,” bupropion, and bupropion + SSRI (HRs 0.28-0.74). Psychotherapy alone (without medication) had a lower self-harm risk than no treatment (HR 0.56, 95% CI 0.52-0.60; *P*=8.76×10^-58^). The sensitivity analysis showed that the direction of drug-outcome associations did not change as a function of the self-harm probability threshold.

**Conclusions:**

Our data support evidence on the effectiveness of antidepressants, MSAs, and psychotherapy for self-harm prevention in BD.

**Trial Registration:**

ClinicalTrials.gov NCT02893371; https://clinicaltrials.gov/ct2/show/NCT02893371

## Introduction

Self-harming behavior is a public and mental health concern of increasing prevalence, which contributes to US hospitalization rates, morbidity, and mortality due to completed suicides. There is a clear temporal and causal link between self-injury and suicide attempts, with both being part of a “suicidality” spectrum and the former being a robust prospective predictor of the latter [1]. In 2018, suicide was the 10th leading cause of death in the general US population, reaching a rate of 14.2 per 100,000 standard population [2]. A previous study reported that the risk ratio of suicide in mental disorders was as high as 7.5 (95% CI 6.6-8.6) and in mood disorders was even higher at 12.3 (95% CI 8.9-17.1) [3]. A recent systematic review showed that bipolar disorder (BD) may be associated with the highest suicide risk among all psychiatric disorders, with over 15%-20% of deaths attributed to suicide and the standardized suicide rate being 20 to 30-fold greater than in the general population (0.2-0.4 per 100 person-years) [4]. Another review found that up to 20% of individuals with BD end their life by suicide and 20%-60% attempt suicide at least once in their lifetime [5]. The reported proportion of suicide attempts and completed suicides among individuals with BD varies from 5:1 in males over 45 years to 85:1 in females under 30 years [6].

Since suicide is an extreme form of self-harming behavior, proper recognition and management of patients presenting with self-inflicted injury are of tremendous importance to prevent lethal outcomes, especially among patients with mood disorders. The factors affecting self-harm risk should be of particular importance for studying suicidality, especially given that the self-inflicted nature of physical trauma/poisoning is often hidden owing to poor patient rapport, provider screening, and data recording.

Incomplete suicidality coding in administrative claims data is a known obstacle for observational studies. It was shown that only 19% of suicide attempts mentioned in primary care clinical notes were coded in International Classification of Diseases-9-Clinical Modification (ICD-9-CM) [7]. Our data from a large-scale observational study on imputing self-harm phenotypes in individuals with major mental illness (MMI) showed that only 1 in 19 self-harm events were coded in the billing records [8]. In addition, a methodological challenge is that ICD-9-CM coding does not robustly distinguish between suicide attempts (implying a desire to die), self-inflicted injury without suicidal intention, and suicide. While ICD-10-CM can distinguish these, many suicide attempts will be classified only under intentional self-harm. Given that all these acts are within the spectrum of self-damaging behavior, we will refer to them collectively as “self-harm.” Thus, we use “self-harm”/“self-harming behavior” as the broadest term covering all forms of self-damaging acts (not thoughts alone), including not only suicide attempts, but also any intentional harm regardless of intent to die. In contrasting this self-harm study with the literature, we recognized that most of the latter was focused more narrowly on attempted and/or completed suicides.

With most of the negative outcomes missing from the data, it is challenging to assess the evidence on treatment strategies for the prevention of self-harm in BD, including pharmacotherapy and psychotherapy. There are conflicting data from studies on the drug-dependent risk of self-harm, and there is still major uncertainty regarding the preventive effect of monotherapy and drug combinations. The benefits of lowering suicidality risk were reported for lithium [9], mood-stabilizing anticonvulsants (MSAs) [10], antidepressants [11-13], and second-generation antipsychotics (SGAs) [14] in the mentally ill population. Several studies demonstrated the benefits of continuous MSA use (either alone or as an adjunct) for suicide risk reduction [15,16]. However, two recent meta-analyses showed no clear benefits of lithium [17] or valproate [18] use for preventing suicidality in patients with mood disorders. The STEP-BD study failed to find any relationship between lithium, MSA, or antipsychotic use and suicidality [19]. The US Food and Drug Administration (FDA) issued warnings for increased suicidality risk with antidepressants [20] and antiepileptic drugs [21].

Two recent meta-analyses showed that psychotherapy is associated with a reduced risk of attempting suicide, but more equivocal evidence on self-harm [22,23]; however, data on psychotherapy-dependent self-harm in adults and subjects with BD are lacking. This provokes further questions on its relative effectiveness when compared with BD medications.

The aim of this study was to provide a comprehensive comparison of all commonly used BD pharmacotherapies, as well as psychotherapy for the risk of self-harm in a large population of commercially insured individuals, using self-harm imputation to overcome the known limitations of this outcome being underrecorded within US electronic health care record systems.

## Methods

A retrospective observational study was conducted using the IBM MarketScan commercial claims and encounters (CCAE) administrative claims data and MarketScan Medicare data on 1.3 million US inpatients and outpatients with BD for the years 2003 to 2016 [24]. The database contained records of provider visits, diagnoses, procedures, outpatient prescription fills, laboratory test orders (but not results), and patient age, sex, and state of residence. The data handling was similar to that in our previous studies on the drug-dependent risk of kidney disorders and diabetes mellitus in BD [25,26], with the additional step of combining data for patients who were covered in both the CCAE and Medicare databases through their patient identifier. The relevant PostgreSQL queries and source code for data transformations and machine learning (ML) are available online [27]. The study protocol was approved by the University of New Mexico Human Research Review Committee (Institutional Review Board number 16-243).

Given that the majority of suicide attempts and self-harm events are not coded at the point of care, we employed ML to build a classification model of self-harm being present or absent, based on billing codes during emergency room (ER) or inpatient provider visits. For that purpose, we constructed a “meta-visit” by merging consecutive outpatient/inpatient/ER visits, with no gaps between visits, which allowed us to capture the medical activity associated with a given event that could have involved multiple points of care. A self-harm phenotype was defined by the presence during a meta-visit of one or more of the ICD-10-CM codes or ICD-9-CM codes listed in Multimedia Appendix 1. These encompass all codes for *intentional* self-harm or suicide attempts by any means, including poisoning. If one or more of these codes was present during a meta-visit, the meta-visit was labeled as class 1; otherwise, it was labeled as class 0.

Our earlier imputation model on over 10 million patients aged ≤65 years with MMI (schizophrenia, schizoaffective disorder, BD, and major depressive disorder) from CCAE was validated with several approaches, including via a clinician-derived “gold standard,” and it identified 10.1 times more self-harm events with probability over 0.5 than were originally coded or 19 times more self-harm events based on summed probabilities [8]. In this study, we applied the previously developed ML modeling approach to an extended set of psychiatric patients of all ages, including those in the CCAE and Medicare databases. We first selected 11 million individuals with any MMI diagnosis (635,722,756 meta-visits) and performed ML on a subset of 26,392,236 meta-visits in which an inpatient or ER visit was present, using five-fold cross-validation. Covariates included age, sex, start year of the meta-visit, and the presence/absence of non–self-harm billing codes. ICD-9-CM and ICD-10-CM diagnosis codes were mapped to their Systematized Nomenclature of Medicine (SNOMED) equivalents (and all ancestors thereof) using the Observational Medical Outcomes Partnership (OMOP) vocabulary as of October 24, 2020 [28]. Procedure codes based on ICD-9-CM Volume 3 (ICD-9-CM V3), ICD-10-Procedure Coding System (ICD-10-PCS), and Current Procedural Technology, Fourth Edition (CPT-4) were mapped to ICD-10-PCS concepts (and all ancestors thereof). Overall, 190,919 covariates were added into the ML process described previously [8]. A threshold probability over 0.5 from the resulting cross-validated model estimates of self-harm was chosen to label self-harm as “present” for our main model, but sensitivity analyses were run for threshold probabilities greater than 0.20, 0.30, 0.40, 0.50, 0.60, 0.70, 0.80, 0.90, 0.95, and only coded self-harm (probability=1.0).

We then used the categorization of 26 million meta-visits to assign whether or not self-harm occurred following treatment exposure in a subset of 529,359 patients with two or more diagnoses of BD and no other MMI, who satisfied our data staging and inclusion/exclusion criteria (see below). Since there are approximately 28 attempts for every suicide death [29] and attempts are a subset of self-harm, selection of self-harm as the outcome allowed us to greatly increase the power of our subsequent comparative effectiveness study.

It should be noted that our ML approach was trained only on meta-visits with an inpatient/ER component since there was a negligible number of self-harm events coded during the purely outpatient meta-visits (about 1 in 100,000).

The patient inclusion criterion was two or more ICD-9-CM/ICD-10-CM diagnostic codes for BD (296.(0-1)*, 296.(4-8)*, F30*, or F31*) from 2003 to 2016. The exclusion criterion was the diagnosis of major depressive disorder, schizophrenia, or schizoaffective disorder at any time during the observation period. The onset of intellectual disability, autism spectrum disorder, mental illness of organic origin, or Parkinson disease, and use of antidementia drugs after the index exposure were considered as censoring events.

**Figure 1:**
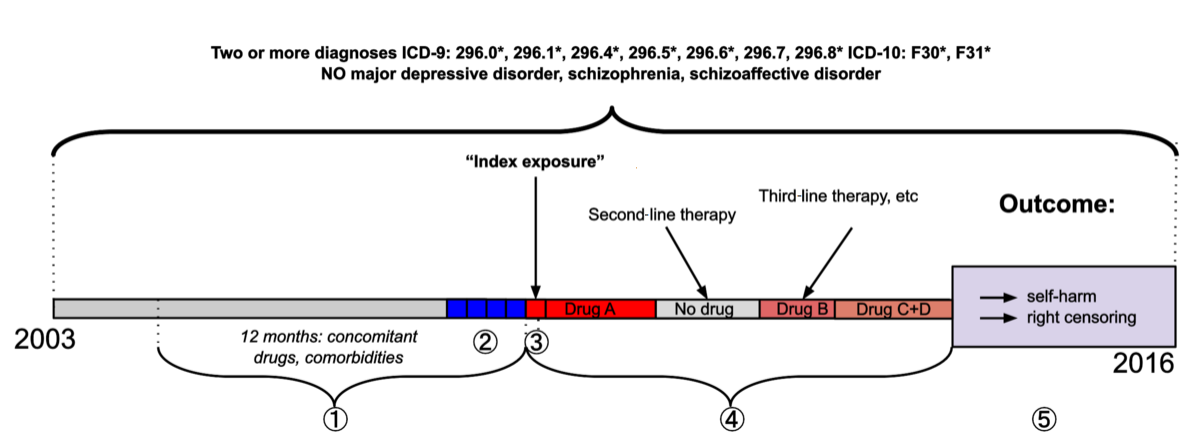
Prespecified sequence of events. (1) One year before the index exposure; (2) Index visit (any meta-visit with a diagnosis of bipolar disorder); (3) Index exposure (the first day of exposure [drugs of interest or no drugs of interest] observable on the last day of the index visit); (4) Time-varying drug exposure period (series of time intervals in which distinct regimens [including “no drug”] were prescribed); (5) Outcome (the first meta-visit with coded and/or imputed self-harm or a censoring event).

A patient was included in the analysis based on the following first observed sequence of events (Figure 1): (1) A minimum of 12 months of observation (used to compute pretreatment covariates); (2) *Index visit* (meta-visit with at least one BD diagnostic code); (3) *Index exposure* (the first day of exposure [drug regimen or “no drug”] observable on the last day of the index visit); (4) *Time-varying drug exposure period* (series of time intervals in which distinct regimens [including “no drug”] were prescribed); and (5) Outcomes of interest (the first meta-visit with newly observed coded and/or imputed self-harm and right censoring defined as any hospitalization/ER meta-visit without coded and/or imputed self-harm, or the end of patient observation).

The observation period ended for patients upon self-harm–unrelated hospitalization/ER meta-visit, because data on pharmacotherapy were not available during these types of visits, making it challenging to quantify psychotropic treatment time intervals. Additionally, hospitalization itself can affect the risk of self-harm.

The start and stop times were recorded for each treatment exposure period. Two Cox regression models for self-harm were built. One model compared 64 pharmacotherapies (as well as “no drug”) to lithium, and the other model compared any drug (as a single category) with or without psychosocial interventions to “no treatment” (neither pharmacotherapy nor psychosocial interventions).

The idea to include “no drug” and “no treatment” in the list of comparators in our study came from patients with BD who participated in several focus groups and were engaged in designing this research [30,31]. Doing so allowed us to address patient questions regarding the safety and effectiveness of avoiding pharmacotherapy.

To ensure sufficient power to detect significant self-harm risk differences and assure convergence of Cox regression, each drug regimen was required to have 1000 or more treatment intervals and to have five or more defined cases of coded and/or imputed self-harm following exposure [32]. Because of this latter restriction, for the sensitivity analyses, the lower threshold sensitivity Cox models will have more drugs analyzed than the higher threshold ones.

The following 11 drug classes were included in the analysis: lithium, first-generation antipsychotics (FGAs), SGAs, third-generation antipsychotics (TGAs; partial agonists of dopamine receptors, aripiprazole, and brexpiprazole), MSAs, monoamine oxidase inhibitor antidepressants, noradrenergic and specific serotonergic antidepressants (NASSAs; represented by mirtazapine only), norepinephrine-dopamine reuptake inhibitors (NDRIs; represented by bupropion only), serotonin-norepinephrine reuptake inhibitors (SNRIs), selective serotonin reuptake inhibitors (SSRIs), and tri- and tetracyclic antidepressants (see Multimedia Appendix 2 for the full list of drugs).

MSAs, SGAs, and TGAs were studied as a class when used during a polypharmacy regimen exposure interval and as individual drugs when considering monotherapy time intervals. SGAs common enough for individual analysis were risperidone, olanzapine, quetiapine, ziprasidone, asenapine, paliperidone, and lurasidone. The individual MSAs studied were valproate, carbamazepine, oxcarbazepine, and lamotrigine. Of the two TGAs, only aripiprazole was common enough to be studied individually.

Combinations of two, three, or four of the 11 drug classes (represented usually by one drug from each class) with the requisite 1000 or more treatment intervals and five or more self-harm events were included in the regression model, and drug regimens without those requisites were grouped under the categories “polypharmacy 2,” “polypharmacy 3,” and “polypharmacy 4” (for uncommon combinations of two, three, and four or more classes, respectively). Enough instances of within-class polypharmacy were present among MSAs and SGAs to include “multi-MSA” and “multi-SGA” variables. Monotherapies without the requisite 1000 exposure intervals (clozapine, brexpiprazole, and iloperidone) were combined into the category “uncommon monotherapy.”

Treatment in the main time-varying Cox regression model was represented as one or more exposure intervals, with all drug categories mutually exclusive and collectively exhaustive, using lithium monotherapy as the reference. The rules to distinguish between polypharmacy and overlapping drug regimen switch are described in our previous study of a similar design [25].

Among the covariates included in the main Cox regression model (not to be confused with the ML covariates) were patient age, sex, BD episode index visit characteristics (severity, mood polarity, and psychotic features, if documented), comorbid mental and physical conditions, including “external injury” codes evidencing noniatrogenic trauma, medication prescriptions filled (other than drugs of interest) and mental health procedures performed 1 year before (but not including) the index exposure, hospital/ER admissions 1 year prior to the index exposure, and types of visits composing the index meta-visit (inpatient/ER/outpatient).

Patient age and the number of unique BD drugs previously tried by the patient were fitted in both Cox regression models using a smoothing spline to account for nonlinear risk of self-harm.

“Psychotherapy” included 227 procedure codes indicating psychosocial intervention (individual, group, or family psychotherapy, crisis intervention, substance abuse–focused treatment, hypnosis, biofeedback, etc) [27].

We developed two time-varying Cox regression models. In the first (main) model comparing 64 treatments and “no drug” to lithium, psychotherapy was coded as a binary time-varying covariate (indicating whether at least one of the 227 procedure codes was present during the current drug/“no drug” exposure period). In the second regression model, all drug regimens were united into a single category (“pharmacotherapy”), and psychotherapy was combined with pharmacotherapy in a time-varying covariate with the following four categories: “pharmacotherapy alone,” “psychotherapy alone,” “psychotherapy and pharmacotherapy,” and “no psychotherapy and no pharmacotherapy” (ie, “no treatment”), with “no psychotherapy and no pharmacotherapy” as the reference.

Given the multiple treatment comparators chosen and the time-varying nature of the treatment covariates in our design, propensity score matching was not feasible for bias correction. Instead, we used a resolution IV fractional factorial design of experiments [33] (whereby main effects are aliased with three-way interactions and two-way interactions are aliased with two-way interactions) to select an appropriate subset of the 78 pretreatment covariates to control for bias. Rather than assessing whether the pretreatment covariates were associated with the outcome, we assessed in a form of sensitivity analysis whether their inclusion or exclusion impacted the hazard ratio (HR) estimates for the treatments with respect to the outcome. If so, inclusion of the variable in the model would be needed for addressing bias. If not, the variable, while possibly associated with the outcome, would nevertheless be unimportant for accurate assessment of treatment risk, and could be excluded to reduce the degrees of freedom of the model and thereby increase power. The time-varying treatment variables were included in each model, but the pretreatment covariates were included or excluded according to the factorial design across 512 different runs (plus a reference run with no pretreatment covariates) to determine which covariates had the largest impact on the drug HR coefficients. The 513 runs generated a 513×66 matrix *Y* of coefficients for 66 drugs over the 513 runs. The design matrix *X* was a 513×78 matrix of +1/−1 values corresponding to whether the given pretreatment covariate was included/excluded in a given run. Then, for each of the 66 column vectors (*Y*_i_) of *Y*, a multiple linear regression was run with *Y*_i_ as the dependent variable and the 78 column vectors of *X* as the independent variables. We counted how many times each of the 78 covariates was significant at *P*<.05/66 over those 66 models to rank candidate covariates for our model. We discarded 26 covariates that were not significant in any of the 66 models. We then built our main Cox regression model using this set of covariates plus the treatment covariates and performed a backward elimination procedure on the pretreatment covariates, iteratively dropping the covariates that were significant in the fewest models and stopping the elimination procedure when a highly significant covariate was found (neoplasm). One drug was subsequently removed from the analysis owing to lack of events when some coding errors were corrected. We also generated an L2-norm of each row of *X* with the reference run row to form a vector *Y*’ for regression with the design matrix *X* to assess how much the incorporation of pretreatment covariates changed all drug covariate estimates in order to understand the largest potential sources of bias. The final set of covariates selected for the first Cox model was used in the second Cox model.

The study used the following software: PostgreSQL version 10.4 (PostgreSQL Global Development Group) and R version 3.4.0 (R Foundation for Statistical Computing), including the Cox regression coxph() function from the survival (2.42-6) package and the FrF2 (1.7-2) package for fractional factorial design. All hypothesis tests were two-sided.

## Results

The following self-harm classification results were observed for our MMI ML model on meta-visits based on five-fold cross-validation (probability [p] cutoff of 0.5): self-harm coded and imputed (p>0.5; N=93,311); self-harm coded but not imputed (p≤0.5; N=3717); self-harm not coded but imputed (N=1,029,058); and self-harm neither coded nor imputed (N=25,266,150) (area under the curve [AUC]=0.99; Matthews correlation coefficient [MCC]=0.28; sensitivity=0.962; specificity=0.961). The following self-harm classification results were observed when the model was applied to meta-visits for only BD cases meeting our eligibility criteria: self-harm coded and imputed (p>0.5; N=488); self-harm coded but not imputed (p≤0.5; N=37); self-harm not coded but imputed (N=8288); and self-harm neither coded nor imputed (N=520,546) (AUC=0.994; MCC=0.225; sensitivity=0.930; specificity=0.984). Thus, an extra 8288 meta-visits with imputed self-harm were added to our analytical pipeline in addition to the 525 (488+37) meta-visits that had coded self-harm for a total of 8813 persons with self-harm.

The sample sizes at different stages of the study are shown in Multimedia Appendix 3. A total of 529,359 patients met the eligibility criteria and had the prespecified sequence of events. Of them, 98.3% were censored and 1.66% (n=8813 events) had imputed and/or coded self-harm.

During the observation period after the index visit, the annual incidence of self-harm (p>0.5) was 0.013 (0.016 for all drug exposure intervals with or without psychotherapy and 0.011 for “no drug” intervals with or without psychotherapy), based on 632,512 years of observation. By summing the probabilities, during the observation period after the index visit for all exposures, the annual incidence of self-harm was 0.027 over 632,512 years of patient observation.

The 515 observed treatment regimens were collapsed to 17 monotherapies, three monoclass therapies, “no drug,” and 45 drug combinations that fit the selection criteria.

The first Cox regression model comparing 65 treatment regimens to lithium showed that 11 treatments had a significantly higher risk of self-harm (*P*<.05, no multiple testing correction) (Table 1). The top “high-risk” treatments were “tri/tetracyclic antidepressants + SGA” (HR 2.33, 95% CI 1.28-4.26; *P*=5.73×10^-3^), “SSRI + FGA” (HR 2.26, 95% CI 1.16-4.38; *P*=1.61×10^-2^), “FGA + MSA” (HR 1.82, 95% CI 1.15-2.89; *P*=1.12×10^-2^), and FGA monoclass therapy (HR 1.69, 95% CI 1.19-2.39; *P*=3.20×10^-3^).

Nine regimens had significantly lower risk of self-harm over lithium alone (*P*<.05, no multiple testing correction), including monotherapies with MSAs valproate (HR 0.71, 95% CI 0.61-0.84; *P*=4.57×10^-5^) and lamotrigine (HR 0.74, 95% CI 0.65-0.85; *P*=1.13×10^-5^), SGAs risperidone (HR 0.68, 95% CI 0.56-0.83; *P*=1.82×10^-4^) and aripiprazole (HR 0.70, 95% CI 0.59-0.84; *P*=9.40×10^-5^), and antidepressant classes SNRI (HR 0.65, 95% CI 0.51-0.83; *P*=5.51×10^-4^), SSRI (HR 0.61, 95% CI 0.53-0.71; *P*=6.05×10^-11^), and NDRI (bupropion) (HR 0.50, 95% CI 0.39-0.65; *P*=1.18×10^-7^), as well as the combination of NDRI with SSRI (HR 0.28, 95% CI 0.13-0.60; *P*=1.0×10^-3^) and the “no drug” regimen.

Of the 11 polypharmacy regimens with risk significantly different from that of lithium, only bupropion + SSRI had lower risk (HR 0.28, 95% CI 0.13-0.60; *P*=1.00×10^-3^). Nine of the remaining 10 high-risk polypharmacy regimens contained an antipsychotic (either SGA or FGA, or both), with the exception being lithium + MSA (HR 1.35, 95% CI 1.09-1.67; *P*=5.32×10^-3^). The “no drug” exposure intervals were associated with a significantly lower risk of subsequent self-harm versus lithium monotherapy (HR 0.56, 95% CI 0.50-0.63; *P*=2.79×10^-22^).

To correct for multiple comparisons, we used the Benjamini-Yekutieli procedure to reduce the false discovery rate. This correction yielded 15 regimens with a statistically significant different risk of self-harm versus lithium at a 5% false-discovery rate (which corresponded to a *P* value cutoff ≤.012). Six of them were of higher risk (tri/tetracyclic antidepressants + SGA, FGA + MSA, FGA, SNRI + SGA, lithium + MSA, and lithium + SGA) and nine were of lower risk than lithium (lamotrigine, valproate, risperidone, aripiprazole, SNRI, SSRI, “no drug,” bupropion, and bupropion + SSRI).

**Figure 2:**
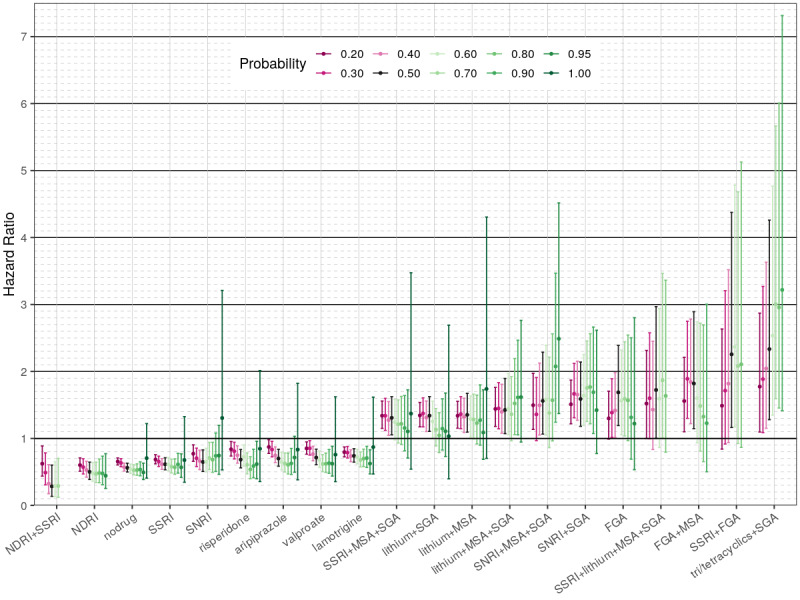
Sensitivity analysis for the “low-risk” and “high-risk” covariates in the first regression model comparing individual exposure regimens for the risk of self-harm. The X-axis shows 13 covariates and the respective 20%-100% self-harm thresholds chosen to impute the outcome. The Y-axis shows the respective hazard ratios (colored dots) and 95% CIs (colored lines). Varied intensity magenta is used to represent the range of 20%-40% self-harm probability thresholds, black is used to represent the 50% threshold of the main model, and varied intensity green is used to represent the 60%-100% probability threshold used. Missing estimates are due to lack of sufficient outcomes for a regimen to be included (observed in the higher probability threshold models). MSA: mood-stabilizing anticonvulsant; NDRI: norepinephrine-dopamine reuptake inhibitor; nodrug: period free from any of the studied bipolar disorder drugs; SGA: second-generation antipsychotic; SNRI: serotonin-norepinephrine reuptake inhibitor; SSRI: selective serotonin reuptake inhibitor.

Our sensitivity analysis revealed that overall most of the “high-risk” drug regimens maintained their HR values above 1 across a wide range of self-harm probability thresholds (40%-70%) (Figure 2). Only one regimen (tri/tetracyclic antidepressants + SGA) demonstrated significantly higher risk of self-harm versus lithium, across all 10 tested probability thresholds.

**Table 1:** Cox regression model comparing 64 pharmacotherapies and “no drug” to lithium for the risk of subsequent coded and/or imputed self-harm in patients with bipolar disorder of all ages.

Covariates^a^	HR^b,c^	Lower 95%	Upper 95%	*P* value	Patients (N=529,359)	Intervals (N=1,749,468)	Events (N=8813)
Tri/tetracyclic antidepressants + SGA^d,e^	2.33	1.28	4.26	5.73×10^-3^	180	1044	11
SSRI^f^ + FGA^g,e^	2.26	1.16	4.38	1.61×10^-2^	195	1014	9
FGA + MSA^h,e^	1.82	1.15	2.89	1.12×10^-2^	481	2448	19
SSRI + lithium + MSA + SGA^e^	1.72	1.00	2.97	4.96×10^-2^	192	1424	14
FGA monoclass therapy^e^	1.69	1.19	2.39	3.20×10^-3^	1069	5853	35
SNRI^i^ + SGA^e^	1.59	1.18	2.14	2.27×10^-3^	1466	6803	50
SNRI + MSA + SGA^e^	1.56	1.06	2.29	2.25×10^-2^	805	4200	29
Asenapine	1.50	0.77	2.90	2.31×10^-1^	160	1494	9
Lithium + MSA + SGA^e^	1.42	1.07	1.90	1.52×10^-2^	1143	7748	57
Lurasidone	1.42	0.97	2.07	7.25×10^-2^	459	3919	29
NDRI^j^ + SSRI + MSA + SGA	1.40	0.66	2.96	3.84×10^-1^	170	1251	7
SSRI + lithium + SGA	1.39	0.94	2.05	9.85×10^-2^	725	3898	28
NDRI + lithium + MSA	1.38	0.65	2.93	3.95×10^-1^	193	1438	7
Aripiprazole + MSA + SGA	1.38	0.86	2.22	1.87×10^-1^	411	2641	18
Lithium + MSA^e^	1.35	1.09	1.67	5.32×10^-3^	2763	18,728	116
Lithium + SGA^e^	1.34	1.11	1.62	2.86×10^-3^	3757	18,980	155
Polypharmacy 4^k^	1.33	0.97	1.82	7.60×10^-2^	1274	8867	47
SSRI + MSA + SGA^e^	1.31	1.05	1.62	1.52×10^-2^	3153	16,322	118
NDRI + MSA + SGA	1.25	0.86	1.81	2.47×10^-1^	836	5558	30
Aripiprazole + SGA	1.22	0.79	1.88	3.72×10^-1^	715	3987	22
NASSA^l^ + SGA	1.20	0.62	2.33	5.86×10^-1^	321	1457	9
NDRI + lithium	1.16	0.74	1.80	5.18×10^-1^	728	4559	21
Tri/tetracyclic antidepressants	1.15	0.71	1.88	5.66×10^-1^	693	4130	17
SSRI + lithium + MSA	1.10	0.66	1.82	7.10×10^-1^	499	3,514	16
Uncommon monotherapy	1.09	0.49	2.45	8.29×10^-1^	169	1205	6
NDRI + SSRI + MSA	1.08	0.68	1.72	7.44×10^-1^	720	4754	19
SSRI + lithium	1.06	0.81	1.39	6.61×10^-1^	2457	13,235	64
MSA + SGA	1.05	0.91	1.21	5.15×10^-1^	13,348	67,185	421
Lithium (reference)	1.00	N/A^m^	N/A	N/A	13,759	66,760	351
Polypharmacy 3^n^	1.00	0.79	1.25	9.66×10^-1^	3794	23,234	95
NDRI + aripiprazole + MSA	0.99	0.51	1.91	9.67×10^-1^	254	2316	9
Lithium + aripiprazole + MSA	0.97	0.50	1.89	9.30×10^-1^	227	1797	9
SNRI + lithium	0.97	0.54	1.72	9.06×10^-1^	529	2814	12
SSRI + SGA	0.96	0.80	1.15	6.36×10^-1^	7896	33,503	188
SSRI + MSA	0.95	0.81	1.11	5.32×10^-1^	12,315	58,789	286
Quetiapine	0.95	0.82	1.10	5.03×10^-1^	13,795	60,422	342
Aripiprazole + MSA	0.94	0.76	1.17	5.90×10^-1^	3401	21,368	108
Ziprasidone	0.94	0.71	1.24	6.63×10^-1^	2381	11,773	58
Multi-SGA	0.93	0.56	1.57	7.94×10^-1^	677	3479	15
Lithium + aripiprazole	0.92	0.60	1.42	7.19×10^-1^	665	4268	22
SNRI + MSA	0.90	0.68	1.20	4.80×10^-1^	2939	13,995	58
FGA + lithium	0.89	0.40	1.99	7.73×10^-1^	315	1472	6
NDRI + SGA	0.89	0.62	1.27	5.07×10^-1^	1408	8336	33
Multi-MSA	0.87	0.61	1.24	4.49×10^-1^	1451	8792	34
SSRI + aripiprazole	0.85	0.64	1.14	2.83×10^-1^	2285	11,695	52
Olanzapine	0.84	0.66	1.07	1.57×10^-1^	4040	16,759	83
NASSA + MSA	0.82	0.42	1.60	5.65×10^-1^	438	2313	9
NDRI + MSA	0.82	0.64	1.05	1.20×10^-1^	3460	21,294	74
Oxcarbazepine	0.81	0.65	1.02	6.91×10^-2^	4436	20,633	97
Carbamazepine	0.74	0.54	1.02	6.45×10^-2^	2284	10,662	42
Lamotrigine^e^	0.74	0.65	0.85	1.13×10^-5^	28,624	131,786	549
NDRI + aripiprazole	0.73	0.41	1.30	2.91×10^-1^	564	4038	12
Valproate^e^	0.71	0.61	0.84	4.57×10^-5^	14,718	61,544	253
SSRI + aripiprazole + MSA	0.70	0.43	1.15	1.56×10^-1^	784	5148	17
Aripiprazole^e^	0.70	0.59	0.84	9.40×10^-5^	8872	47,373	186
Polypharmacy 2^o^	0.68	0.46	1.01	5.32×10^-2^	2017	11,269	28
Risperidone^e^	0.68	0.56	0.83	1.82×10^-4^	7084	28,302	138
SNRI^e^	0.65	0.51	0.83	5.51×10^-4^	6120	27,921	78
NASSA	0.65	0.38	1.08	9.77×10^-2^	950	4964	15
Paliperidone	0.61	0.29	1.30	2.03×10^-1^	292	1858	7
SSRI^e^	0.61	0.53	0.71	6.05×10^-11^	30,138	131,895	381
NDRI + SSRI + SGA	0.60	0.25	1.45	2.57×10^-1^	345	2079	5
“No drug”^e^	0.56	0.50	0.63	2.79×10^-22^	299,295	621,467	3694
NDRI (bupropion)^e^	0.50	0.39	0.65	1.18×10^-7^	6005	35,433	72
SNRI + aripiprazole	0.45	0.19	1.09	7.85×10^-2^	457	2765	5
NDRI (bupropion) +SSRI^e^	0.28	0.13	0.60	1.00×10^-3^	1263	7496	7
Prior self-harm^e^	3.32	2.68	4.11	3.17×10^-28^	704	1652	70
Alcohol/substance abuse or dependence^e^	1.92	1.81	2.03	7.17×10^-106^	57,392	149,679	1065
Delirium^e^	1.69	1.36	2.10	1.92×10^-6^	1694	4086	60
Prior hospitalization^e^	1.63	1.54	1.72	1.78×10^-64^	131,613	342,122	1905
Mental procedure before index exposure^e^	1.50	1.42	1.58	3.43×10^-50^	81,943	247,474	841
Liver disease^e^	1.49	1.29	1.71	4.27×10^-8^	9063	23,559	121
Unknown polarity of index mood episode^e^	1.33	1.24	1.43	1.67×10^-15^	307,243	1,000,348	2447
Conduct disorder^e^	1.26	1.14	1.39	3.65×10^-6^	13,728	39,657	255
Seizure disorder^e^	1.18	1.11	1.25	2.07×10^-7^	90,276	307,887	667
External injury^e^	1.12	1.06	1.19	2.31×10^-5^	117,722	338,635	1350
Pulmonary disorder	1.12	0.99	1.26	7.60×10^-2^	18,960	48,974	160
Depression during the index meta-visit^e^	1.10	1.02	1.18	1.52×10^-2^	72,386	245,707	487
Male sex^e^	1.07	1.03	1.12	1.46×10^-3^	227,507	733,963	1966
Exposure to sedative or antianxiety drug^e^	1.06	1.00	1.12	4.24×10^-2^	130,186	431,040	929
Number of prior unique BD^p^ drugs tried (linear component of spline fit)^e^	1.02	1.01	1.04	4.17×10^-3^	N/A	N/A	N/A
Psychotic features present during the index meta-visit	1.01	0.92	1.11	7.73×10^-1^	23,846	78,579	345
Age (linear component of spline fit)^e^	0.98	0.97	0.98	6.99×10^-201^	N/A	N/A	N/A
Exposure to central nervous system stimulant	0.95	0.88	1.01	9.62×10^-2^	55,140	195,116	396
BD type II during the index meta-visit^e^	0.93	0.87	0.99	2.49×10^-2^	90,741	331,720	497
Manic episode during the index meta-visit^e^	0.92	0.85	0.99	3.40×10^-2^	82,955	250,285	512
Exposure to glucocorticoids^e^	0.83	0.77	0.89	1.04×10^-7^	80,626	246,182	472
Exposure to antibacterial agents^e^	0.80	0.76	0.84	3.77×10^-17^	144,933	453,129	951
Exposure to sex hormones^e^	0.79	0.74	0.85	3.08×10^-10^	67,550	217,335	372
Neoplasm^e^	0.77	0.70	0.85	1.70×10^-7^	42,628	130,488	220
Psychotic features unknown during the index meta-visit^e^	0.67	0.63	0.72	2.85×10^-30^	401,789	1,295,408	2709
Psychotherapy (psychosocial interventions)^e^	0.59	0.57	0.62	1.12×10^-114^	249,328	704,937	1369
Outpatient visit present during the index meta-visit^e^	0.56	0.50	0.63	4.69×10^-23^	522,232	1,732,715	3475
Age (nonlinear components of the spline model)^e^	N/A	N/A	N/A	4.58×10^-32^	N/A	N/A	N/A
Number of prior unique BD drugs tried (nonlinear components of spline fit)^e^	N/A	N/A	N/A	1.21×10^-9^	N/A	N/A	N/A

^a^Covariates labeled “prior” are related to the 1-year period before the index exposure.

^b^Covariates are sorted by their hazard ratio value.

^c^HR: hazard ratio.

^d^SGA: second-generation antipsychotic.

^e^Covariates with significant *P* values (<.05; no multiple testing correction).

^f^SSRI: selective serotonin reuptake inhibitor.

^g^FGA: first-generation antipsychotic.

^h^MSA: mood stabilizing anticonvulsant.

^i^SNRI: serotonin-norepinephrine reuptake inhibitor.

^j^NDRI: norepinephrine-dopamine reuptake inhibitor (represented by bupropion only).

^k^Polypharmacy 4: uncommon combination of four or more bipolar disorder drug classes.

^l^NASSA: noradrenergic and specific serotonergic antidepressant (represented by mirtazapine only).

^m^N/A: not applicable.

^n^Polypharmacy 3: uncommon combination of three bipolar disorder drug classes.

^o^Polypharmacy 2: uncommon combination of two bipolar disorder drug classes.

^p^BD: bipolar disorder.

For most of the “low-risk” drugs, the HR values were below 1 at any self-harm probability threshold, except for 90%-100% (very likely to be self-harm or actually coded). Bupropion alone or in combination with SSRI had a significant association with lower self-harm risk across all tested thresholds. As expected, the higher the probability of self-harm, the larger were the respective HR CIs owing to fewer events observed. The results of the sensitivity analysis for all exposure covariates in this model, as well as the nondrug covariates, can be found in Multimedia Appendix 4, Multimedia Appendix 5, Multimedia Appendix 6, and Multimedia Appendix 7.

When assessing the largest sources of bias, four variables were highly significantly associated with shifting the estimates of all the treatment coefficients, based on the regression of *Y’* versus *X*, including the number of prior unique BD drugs tried, psychotherapy, alcohol/substance abuse or dependence, and outpatient visit present during the index meta-visit. These four were among the covariates with the top five most significant (*P*<.05/66) associations over the 66 *Y_i_* versus *X* regressions performed on individual treatment estimates in our variable selection procedure. A total of 29 pretreatment covariates were incorporated in the model to adjust for potential bias in treatment risk estimates.

In the main Cox model, documentation of prior coded self-harm had the highest HR value among all nondrug covariates (HR 3.32, 95% CI 2.68-4.11; *P*=3.17×10^-28^). A set of mental conditions, including delirium, substance/alcohol abuse and dependence, conduct disorder, and procedures related to mental health services were associated with a significantly higher risk of self-harm (HR 1.26-1.92, *P*<.05). Previous hospitalizations, liver disease, and seizures were also associated with elevated self-harm risk when present (HR 1.18-1.63, *P*<.05). Exposure to antianxiety and sedative drugs showed a modest risk of self-harm (HR 1.06, 95% CI 1.00-1.12; *P*=4.24×10^-2^). Additionally, index visit depression was modestly associated with self-harm risk (HR 1.10, 95% CI 1.02-1.18; *P*=1.52×10^-2^).

Multiple factors had significantly lower self-harm risk, including index manic mood episodes, BD type II, use of antibacterial agents and glucocorticoids, exposure to sex hormones, and neoplasm diagnosis (HR 0.77-0.93; *P*<.05). Psychotherapy during the exposure period was strongly associated with a lower risk of self-harm (HR 0.59, 95% CI 0.57-0.62; *P*=1.12×10^-114^). The lowest HR value for self-harm was associated with an outpatient visit being present during the index meta-visit (HR 0.56, 95% CI 0.50-0.63; *P*=4.69×10^-23^) (Table 1).

**Figure 3:**
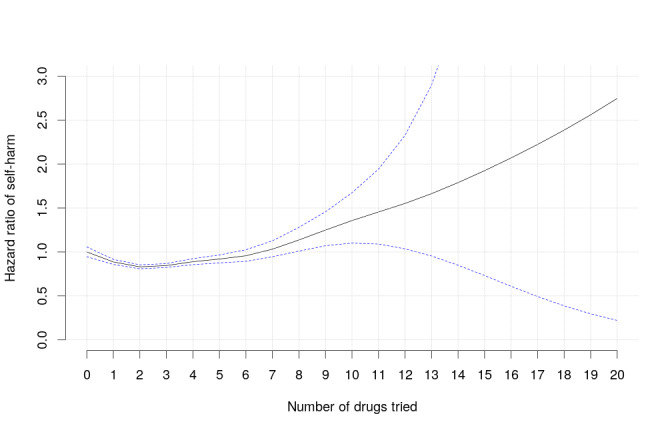
The hazard ratio of coded and/or imputed self-harm as a function of the number of different unique drugs of interest used by the patient in the year prior to the index visit plus up to the prior treatment interval. The graph represents a smoothing spline, with the reference being zero prior drugs. The blue dotted lines represent 95% CIs.

When self-harm risk was plotted as a function of the number of different unique drugs of interest tried in the past, we observed that HR values slightly decreased after intervals with one and two drugs used, but then started to rise with the number of agents used (Figure 3).

**Figure 4:**
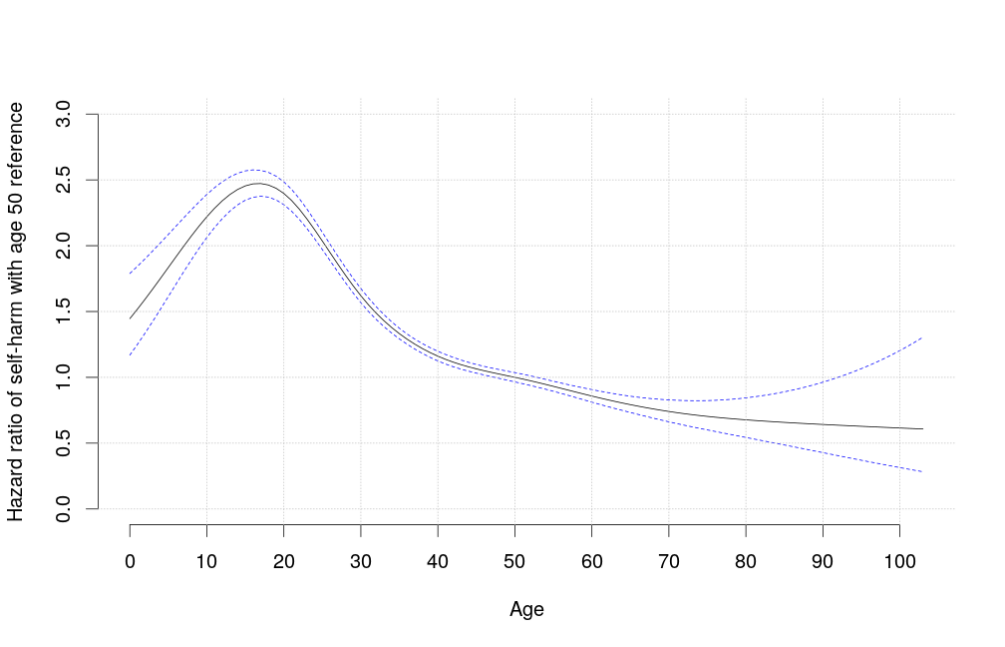
The hazard ratio of coded and/or imputed self-harm as a function of patient age. The graph represents a smoothing spline, with the reference being age 50 years. The blue dotted lines represent 95% CIs.

Figure 4 shows the risk of self-harm as a function of patient age. It demonstrates that HR values were much higher in adolescence, dropped after the 20s, and leveled off with older age.

In the second Cox regression model with all BD drugs grouped under the “pharmacotherapy” category, the risk of self-harm was the lowest following “psychotherapy alone” intervals, compared with “no treatment” (HR 0.56, 95% CI 0.52-0.60; *P*=8.76×10^-58^) (Table 2). The combination “psychotherapy + pharmacotherapy” had a somewhat lower risk of self-harm (HR 0.88, 95% CI 0.83-0.95; *P*=3.80×10^-4^), but pharmacotherapy alone was associated with a significantly higher risk compared with “no treatment” (HR 1.38, 95% CI 1.30-1.48; *P*=1.09×10^-22^).

**Table 2:** Cox regression model comparing “pharmacotherapy” (as a single category) and “psychotherapy” (psychosocial interventions) to “no treatment” (no drugs and no psychotherapy) for the risk of subsequent coded and/or imputed self-harm in patients with bipolar disorder of all ages.

Covariates^a^	HR^b,c^	Lower 95%	Upper 95%	*P* value	Patients (N=529,359)	Intervals (N=1,749,468)	Events (N=8813)
Pharmacotherapy alone (any drug regimen)^d^	1.38	1.30	1.48	1.09×10^-22^	122,898	651,191	2819
Any drug and psychotherapy^d^	0.88	0.83	0.95	3.80×10^-4^	107,166	476,810	2300
Psychotherapy alone^d^	0.56	0.52	0.60	8.76×10^-58^	142,162	228,127	1183
No treatment (reference)	1.00	N/A^e^	N/A	N/A	157,133	393,340	2511
Prior self-harm^d^	3.32	2.68	4.10	3.34×10^-28^	704	1652	70
Alcohol/substance abuse or dependence^d^	1.91	1.80	2.03	2.48×10^-105^	57,392	149,679	1065
Delirium^d^	1.68	1.35	2.08	2.74×10^-6^	1694	4086	60
Previous hospitalization^d^	1.61	1.52	1.71	3.21×10^-62^	131,613	342,122	1905
Prior mental health procedure^d^	1.49	1.41	1.57	2.90×10^-49^	81,943	247,474	841
Liver disease^d^	1.48	1.29	1.71	4.30×10^-8^	9063	23,559	121
Unknown polarity of index mood episode^d^	1.35	1.25	1.45	2.33×10^-16^	307,243	1,000,348	2447
Conduct disorder^d^	1.25	1.13	1.38	9.03×10^-6^	13,728	39,657	255
Seizure disorder^d^	1.17	1.10	1.25	4.56×10^-7^	90,276	307,887	667
Pulmonary disorder	1.12	0.99	1.26	6.69×10^-2^	18,960	48,974	160
External injury	1.12	1.06	1.18	4.79×10^-5^	117,722	338,635	1350
Depression during the index visit^d^	1.09	1.01	1.17	3.15×10^-2^	72,386	245,707	487
Male sex^d^	1.08	1.04	1.13	3.96×10^-4^	227,507	733,963	1966
Number of prior unique BD^f^ drugs tried (linear component of spline fit)^d^	1.07	1.06	1.09	2.99×10^-24^	N/A	N/A	N/A
Exposure to sedative antianxiety	1.05	0.99	1.11	1.10×10^-1^	130,186	431,040	929
Psychotic features present	1.03	0.93	1.13	6.01×10^-1^	23,846	78,579	345
Age (linear component of spline fit)^d^	0.98	0.98	0.98	5.17×10^-194^	N/A	N/A	N/A
Exposure to central nervous system stimulant^d^	0.93	0.87	0.99	2.15×10^-2^	55,140	195,116	396
BD type II during the index meta-visit^d^	0.92	0.86	0.98	6.72×10^-3^	90,741	331,720	497
Manic episode during the index meta-visit^d^	0.91	0.84	0.98	1.45×10^-2^	82,955	250,285	512
Exposure to glucocorticoids^d^	0.82	0.77	0.88	2.18×10^-8^	80,626	246,182	472
Exposure to antibacterial agents^d^	0.78	0.74	0.83	2.65×10^-19^	144,933	453,129	951
Exposure to sex hormones^d^	0.78	0.73	0.84	3.77×10^-11^	67,550	217,335	372
Neoplasm^d^	0.76	0.69	0.84	4.76×10^-8^	42,628	130,488	220
Psychotic features unknown during the index meta-visit^d^	0.66	0.62	0.71	8.75×10^-33^	401,789	1,295,408	2709
Outpatient visit present during the index meta-visit^d^	0.56	0.50	0.62	3.19×10^-24^	522,232	1,732,715	3475
Age (nonlinear components of spline model)^d^	N/A	N/A	N/A	3.00×10^-33^	N/A	N/A	N/A
Number of prior unique BD drugs tried (nonlinear components of spline fit)^d^	N/A	N/A	N/A	4.47×10^-12^	N/A	N/A	N/A

^a^Covariates labeled “prior” are related to the 1-year period before the index exposure.

^b^Covariates are sorted by their hazard ratio value.

^c^HR: hazard ratio.

^d^Covariates with significant *P* values (<.05).

^e^N/A: not applicable.

^f^BD: bipolar disorder.

**Figure 5:**
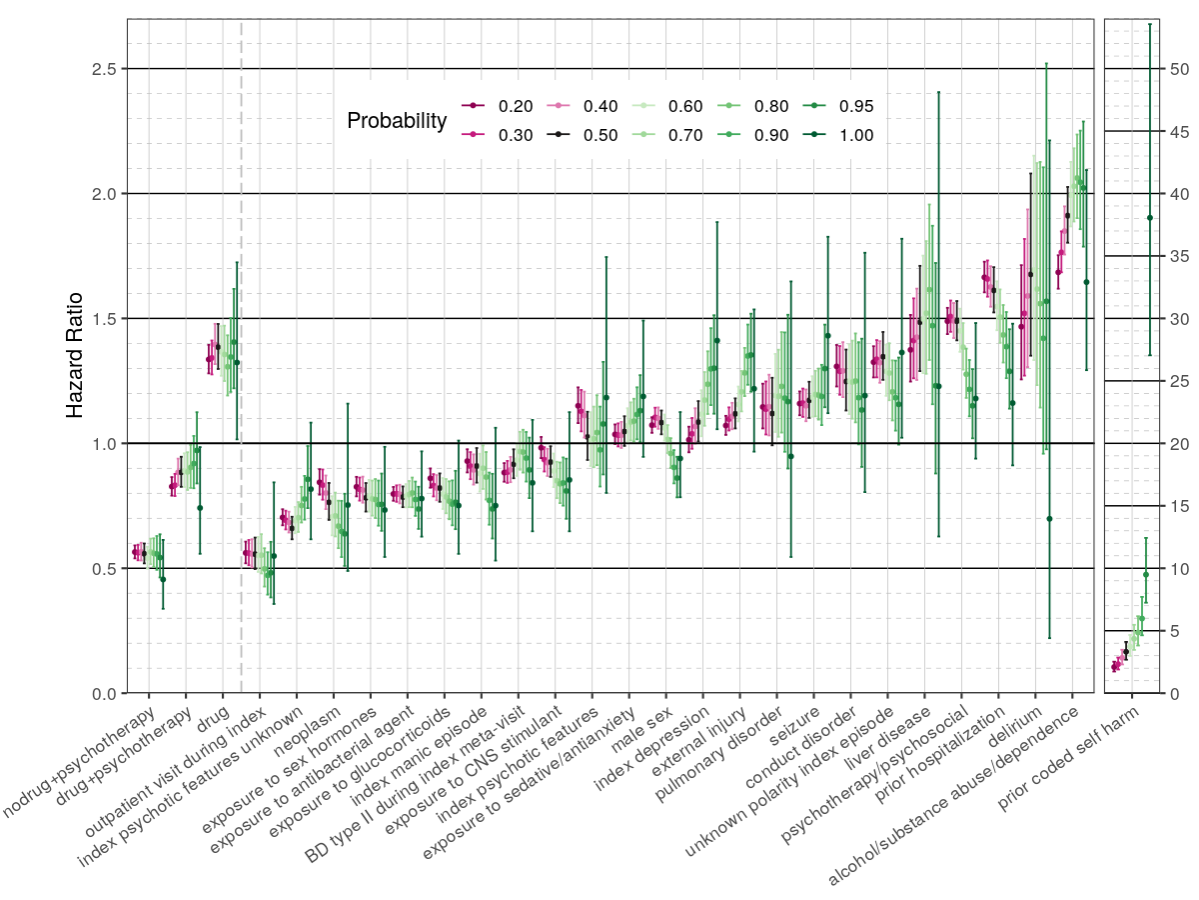
Sensitivity analysis for the “low-risk” and “high-risk” covariates in the second regression model comparing pharmacotherapy (as a single exposure category) and psychotherapy for the risk of self-harm. The X-axis shows 27 covariates and the respective 20%-100% self-harm probability thresholds chosen to impute the outcome. The Y-axis shows the respective hazard ratios (HRs) (colored dots) and CIs (colored lines). Varied intensity magenta is used to represent the range of 20%-40% self-harm probability thresholds, black is used to represent the 50% threshold of the main model, and varied intensity green is used to represent the 60%-100% probability threshold used. The covariate “prior coded self-harm” is separated out with a different HR scale in the far right, since the HR values were extremely high at the 100% (coded) probability threshold. BD: bipolar disorder; CNS: central nervous system; Drug: any of the bipolar disorder drugs of interest.

The sensitivity analysis showed that most of the “high-risk” variables maintained their HR values above 1 at a wide range of self-harm probability thresholds, except for very high thresholds (>80%-90%). Prior self-harm and pharmacotherapy alone (without psychosocial interventions) had significantly high HR values across all tested self-harm probability thresholds. The “low-risk” variables mostly maintained their HR values below 1 with different self-harm probability thresholds (except for 80%-100%). Five variables had significantly lower risk of self-harm across all tested thresholds compared with no treatment at all. They were psychotherapy alone, prior self-harm, outpatient visit present during the index meta-visit, exposure to sex hormones, and use of antibacterial agents. As in the first model, the higher was the probability of self-harm, the wider were the CIs owing to fewer events (Figure 5).

## Discussion

### Principal Findings

Given the use of imputed self-harm (in addition to formally coded) as the primary outcome in our study, it is worthwhile to compare the coded and imputed annual incidence of self-harm in our data with that of the literature. In a recent UK study [34], within 25,965 person-years of observation in a cohort of 6671 patients with pharmacologically treated BD, who were aged 16 years or above, the annual incidence of hospitalized self-harm was 3774 per 100,000 person-years at risk (PYAR). The coded self-harm in our BD cohort of all ages was only 83 per 100,000 PYAR. This would constitute 1:45-fold underrecording, if US rates of self-harm are comparable to UK rates. Our earlier estimate [8] that only 1 in 19 self-harm events was coded (within meta-visits having an inpatient and/or ER component) may not have been sufficiently pessimistic. In contrast to the strikingly low rates of coded self-harm in our study data, the estimates of coded + imputed self-harm used for our main model were more reassuring, with 1393 self-harm events per 100,000 PYAR. When summing the probabilities over all meta-visits, our estimate of the level of self-harm was 2839 per 100,000 PYAR, which is 75% of the UK estimate and is probably still low. Our sensitivity analysis revealed a range of 525 (formally coded only) to 20,226 (coded + imputed with >20% probability) self-harm events corresponding to a range of 83 to 3198/100,000 PYAR. It is important to note that because HRs are relative measures, they may be stably estimated across a broad range of imputation thresholds, with the advantage of more power for lower thresholds.

Our findings suggest that exposure to FGAs and some multidrug combinations were associated with 1.31 to 2.33 higher risks of self-harm compared with lithium; however, these associations were possibly observed owing to multiple-testing type I error. Drug-free intervals (“no drug”) had one of the lowest HR values in our first regression model compared with lithium (HR 0.56, 95% CI 0.50-0.63; *P*=2.79×10^−22^). According to a recent literature review, there is strong converging evidence indicating that long-term lithium treatment lowers deaths by suicide in patients with BD [4], which can be attributed to its possible serotonergic effect [35]. One explanation for the better performance of the “no drug” regimen in our study versus lithium could be indication bias, as drug-free periods can be associated with stable remission or asymptomatic states.

Self-harm risk reduction was significant with monotherapies involving the MSAs valproate and lamotrigine, the atypical antipsychotics risperidone and aripiprazole, the antidepressant bupropion, and monoclass treatment with SNRI and SSRI antidepressants. There are conflicting data in the literature on antidepressant-dependent suicidality in mood disorders, with reports on both the increased [36] and decreased risks of suicidal behavior [11,37]. In 2004, antidepressants received an FDA black box warning owing to increased suicidal thoughts and behaviors in adolescents on antidepressants versus placebo in FDA approval–seeking trials [20], and this warning was extended to include young adults in 2007 [38]. It is still not entirely clear whether a presumed increased suicidality risk in antidepressant users is due to drugs failing to prevent deterioration involving the natural illness course, due to their activating effect, or due to manic switch with subsequent mood phase inversion. In contrast, a 27-year prospective study on mood disorders showed that the risk of suicide attempts or suicides was reduced by 20% among participants taking antidepressants (HR 0.80, 95% CI 0.68-0.95; *P*=.011) [12]. Subsequent findings of the same authors showed that suicidality risk was reduced by 54% in individuals with BD type I and by 35% in those with BD type II while on antidepressants, compared with propensity-matched unexposed intervals [13]. While our study generated evidence on a more broadly defined set of “self-harm” acts, our data support the findings of the relative safety of SSRI and SNRI antidepressants compared with lithium and even with “no drug” in relation to suicidality in BD.

SGAs were previously shown to be associated with a reduced risk of suicide in patients with schizophrenia [14], although two recent international observational studies demonstrated the inferiority of quetiapine and olanzapine compared with lithium for self-harm prevention [34], and even an increased risk of completed suicide among BD patients taking antipsychotics [39]. Our data showed that the SGA risperidone is associated with a significantly lower self-harm risk in patients with BD. There is evidence suggesting that the beneficial effect of antipsychotics in BD may be explained by reduced impulsivity and risk taking [40].

Similar to studies on antidepressants, there are conflicting data on the MSA-dependent risk of self-harm in BD. While some studies reported an equally beneficial effect of MSAs (divalproex and carbamazepine) to lithium for BD suicidality prevention [10], others reported a significantly safer profile for lithium [41]. The majority of MSAs received an FDA warning of increased suicidal thoughts and behaviors in 2008 [42], based on a meta-analysis of 11 drugs [21]. Several studies failed to find any significant changes in suicidality risk according to antiepileptic drug intake [18,37]. However, a large pharmacoepidemiologic study found significantly lower rates of suicide attempts following MSA use, compared with the period before treatment, and showed that MSA monotherapy was significantly protective relative to no pharmacologic treatment (3 per 1000 vs 15 per 1000 person-years) [43]. Our findings support the evidence of a beneficial role of MSAs in self-harm prevention in BD management. Unlike the other data [34], our data showed that valproate is superior to lithium in terms of the association with reduced self-harm risk.

Given that 10 of the 11 “high-risk” exposures in our study were polypharmacy regimens, we made efforts to address the possible indication bias of multidrug regimens being given to patients who are treatment-resistant, by modeling the risk of self-harm as a function of the number of unique BD drugs filled in the year prior to the index visit plus those drugs tried from the index visit up to the current treatment interval. We fit this within the Cox regression model using a smoothing spline with no prior drugs set as the reference (Figure 3). The risk of self-harm was significantly lower in individuals treated with one to five different BD drugs in the year prior to the index visit, compared with individuals who had no prior drugs in the observed period of time. One explanation for this finding is that several “trial and error” attempts eventually result in better control over illness symptoms. However, self-harm risk was significantly higher in patients who received eight or more unique BD drugs, compared with drug-naive subjects, evidencing drug-resistant cases. At the same time, the rapidly expanding range of 95% CI corresponding to 8 to 20 drugs indicates limited sample sizes in this range. Overall, given that our self-harm risk estimates for the drugs account for prior treatment complexity and that the magnitude of this factor’s impact on risk was modest, it seems unlikely that a presumed polypharmacy-dependent increase in self-harm risk in patients with BD is fully explained by drug resistance or disease severity. However, we may not have fully corrected for indication biases. In particular, we did not model drug exposures prior to the year before the index visit.

Our sensitivity analysis showed that the direction of drug-outcome associations did not change as a function of the threshold of self-harm probability, while HR CIs were much more narrow when the outcome was imputed rather than coded. This provides evidence that using ML-imputed outcomes is a promising approach to increase power to perform comparative effectiveness studies, particularly when a phenotype is sparsely coded.

The presence of an outpatient encounter during the index meta-visit (with or without an adjacent hospitalization/ER visit) was associated with the lowest risk of self-harm. This can be explained by more accessible or comprehensive health care services provided, as evidenced by a patient visiting his/her outpatient provider during a crisis.

As was expected, our data suggested that psychosocial interventions may decrease the risk of self-harm in patients with BD. A recent meta-analysis showed that patients who received psychotherapy were less likely to subsequently attempt suicide [22]. However, a surprising finding from our second Cox regression model was that the HR of self-harm was lower following time intervals with psychotherapy alone, rather than when psychotherapy was combined with pharmacotherapy. This could be explained by indication bias, since drug-free patients could be asymptomatic or in stable remission. Another explanation is that pharmacotherapy was a very heterogeneous category combining “low-risk” and “high-risk” regimens together. There was insufficient power to perform a per-drug analysis of adjunctive psychotherapy.

The study limitations include nonrandomized assignment of patients to treatment groups; no patient data availability prior to the insurance enrollment date, as well as prior to 2003; unmeasured indication or other biases (eg, personality traits, coping strategies, environmental stressors, and support systems); and no correction for medication dosage, route of administration, or release mechanism.

### Conclusions

The risk of self-harm varied more than eight-fold among different BD drug regimens. Exposure to antidepressant or MSA monotherapy was associated with a significantly lower risk of subsequent self-harm compared with lithium. Psychotherapy was strongly associated with a decreased risk of self-harm in patients with BD. ML imputation of self-harm can enhance the power for comparative effectiveness studies of BD treatments. The risk of self-harm was the highest during adolescence. Our data support the evidence that prior self-harm is one of the strongest predictors of future self-harm.

## Appendix

Multimedia Appendix 1 List of self-harm billing codes.

Multimedia Appendix 2 Drugs of interest analyzed either individually or as part of a composite class.

Multimedia Appendix 3 Sample size based on data staging. BD: bipolar disorder; ER: emergency room; MMI: major mental illness.

Multimedia Appendix 4 Sensitivity analysis for the first third of drug covariates (sorted by their hazard ratio from low to high) in the regression model comparing individual exposure regimens for the risk of self-harm. The X-axis shows drug covariates and the respective 20%-100% self-harm thresholds chosen to impute the outcome. The Y-axis shows the respective hazard ratios (colored dots) and CIs (colored lines). Varied intensity magenta is used to represent the range of 20%-40% self-harm probability thresholds, black is used to represent the 50% threshold of the main model, and varied intensity green is used to represent the 60%-100% probability threshold used. MSA: mood-stabilizing anticonvulsant; NDRI: norepinephrine-dopamine reuptake inhibitor; SGA: second-generation antipsychotic; SNRI: serotonin-norepinephrine reuptake inhibitor; SSRI: selective serotonin reuptake inhibitor.

Multimedia Appendix 5 Sensitivity analysis for the second third of drug covariates (sorted by their hazard ratio from low to high) in the regression model comparing individual exposure regimens for the risk of self-harm. The X-axis shows drug covariates and the respective 20%-100% self-harm thresholds chosen to impute the outcome. The Y-axis shows the respective hazard ratios (colored dots) and CIs (colored lines). Varied intensity magenta is used to represent the range of 20%-40% self-harm probability thresholds, black is used to represent the 50% threshold of the main model, and varied intensity green is used to represent the 60%-100% probability threshold used. MSA: mood-stabilizing anticonvulsant; NDRI: norepinephrine-dopamine reuptake inhibitor; SGA: second-generation antipsychotic; SNRI: serotonin-norepinephrine reuptake inhibitor; SSRI: selective serotonin reuptake inhibitor.

Multimedia Appendix 6 Sensitivity analysis for the last third of drug covariates (sorted by their hazard ratio from low to high) in the regression model comparing individual exposure regimens for the risk of self-harm. The X-axis shows drug covariates and the respective 20%-100% self-harm thresholds chosen to impute the outcome. The Y-axis shows the respective hazard ratios (colored dots) and CIs (colored lines). Varied intensity magenta is used to represent the range of 20%-40% self-harm probability thresholds, black is used to represent the 50% threshold of the main model, and varied intensity green is used to represent the 60%-100% probability threshold used. MSA: mood-stabilizing anticonvulsant; NDRI: norepinephrine-dopamine reuptake inhibitor; SGA: second-generation antipsychotic; SNRI: serotonin-norepinephrine reuptake inhibitor; SSRI: selective serotonin reuptake inhibitor.

Multimedia Appendix 7 Sensitivity analysis for the nondrug covariates in the regression model comparing individual exposure regimens for the risk of self-harm. The X-axis shows nondrug covariates and the respective 20%-100% self-harm thresholds chosen to impute the outcome. The Y-axis shows the respective hazard ratios (HRs) (colored dots) and CIs (colored lines). Varied intensity magenta is used to represent the range of 20%-40% self-harm probability thresholds, black is used to represent the 50% threshold of the main model, and varied intensity green is used to represent the 60%-100% probability threshold used. The covariate “prior coded self-harm” is separated out with a different HR scale in the far right, since the HR values were extremely high at 100% (coded) probability threshold. MSA: mood-stabilizing anticonvulsant; NDRI: norepinephrine-dopamine reuptake inhibitor; SGA: second-generation antipsychotic; SNRI: serotonin-norepinephrine reuptake inhibitor; SSRI: selective serotonin reuptake inhibitor.

## Abbreviations

AUC: area under curve
BD: bipolar disorder
CCAE: commercial claims and encounters
ER: emergency room
FDA: US Food and Drug Administration
FGA: first-generation antipsychotic
HR: hazard ratio
ICD: International Classification of Diseases
MCC: Matthews correlation coefficient
ML: machine learning
MMI: major mental illness
MSA: mood-stabilizing anticonvulsant
NASSA: noradrenergic and specific serotonergic antidepressant
NDRI: norepinephrine-dopamine reuptake inhibitor
PYAR: person-years at risk
SGA: second-generation antipsychotic
SNRI: serotonin-norepinephrine reuptake inhibitor
SSRI: selective serotonin reuptake inhibitor
TGA: third-generation antipsychotic
