# Supplementary material for: Using Machine Learning Imputed Outcomes to Assess Drug-Dependent Risk of Self-Harm in Patients with Bipolar Disorder: A Comparative Effectiveness Study
Source: JMIR Ment Health. 2021 Apr 21;8(4):e24522. doi: 10.2196/24522 (PMC8100888; doi:10.2196/24522)
Supplement: Multimedia Appendix 1 [file mental_v8i4e24522_app1.docx]

**Multimedia Appendix 1. List of self-harm billing codes.**

| **ICD-9-CM terms used to delineate coded self-harm** |
| --- |
| E95{0-9}* |
| **ICD-10-CM terms used to delineate coded self-harm** |
| X7{1-9}*; X8{0-3}*; T14.91*; T36.{0-8}X2*; T36.92*; T37.{0-8}X2*; T37.92*; T38.{0-7}X2*; T38.8{0,1,9}2*; T38.9{0,9}2*; T39.0{1,9}2*; T39.{1-2}X2*; T39.3{1,9}2*; T39.{4,8}X2*; T39.92*; T40.{0‑5}X2*; T40.6{0,9}2*; T40.{7,8}X2*; T40.9{0,9}2*; T41.{0,1}X2*; T41.2{0,9}2*; T41.{3,5}X2*; T41.42*; T42.{0-6}X2*; T42.72*; T42.8X2*; T43.{0,1,3,4,6,8}X2*; T43.{2,5,6}{0,9}2*; T43.92*; T43.{0,2,6}{1,2}2*; T43.6{3,4}2*; T44.{0-8}X2*; T44.9{0,9}2*; T45.{0-4}X2*; T45.5{1,2}2*; T45.6{0,1,2,9}2*; T45.{7,8}X2*; T45.92*; T46.{0-8}X2*; T46.9{0,9}2*; T47.{0-8}X2*; T47.92*; T48.{0,1}X2*; T48.2{0,9}2*; T48.{3-6}X2*; T48.9{0,9}2*; T49.{0-8}X2*; T49.92*; T50.{0-8}X2*; T50.A{1,2,9}2*; T50.B{1,9}2*; T50.Z{1,9}2*; T50.9{0,1,9}2*; T51.{0-3,8}X2*; T51.92*; T52.{0-4,8}X2*; T52.92*; T53.{0-7}X2*; T53.92*; T54.{0-3}X2*; T54.92*; T55.{0,1}X2*; T56.{0-7}X2*; T56.8{1,9,X}2*; T56.92*; T57.{0-3,8}X2*; T57.92*; T58.{0,1}2*; T58.{2,8}X2*; T58.892*; T58.92*; T59.{0-7}X2*; T59.812*; T59.892*; T59.92*; T60.{0-4,8}X2*; T60.92*; T61.{0,1}2*; T61.7{7,8}2*; T61.8X2*; T61.92*; T62.{0-2,8}X2*; T62.92*; T63.{0,3}02*; T63.{0,1,3-8}12*; T63.92*; T63.{0,1,3,4,6,8}22*; T63.{0,3,4,6,8}32*; T63.{0,4}42*; T63.452*; T63.{0,4}62*; T63.072*; T63.{0,4}82*; T63.{0,1,3,5-8}92*; T63.2X2*; T64.{0,8}2*; T65.2{1,2,9}2*; T65.{0,1,3-6}X2*; T65.8{1-3,9}2*; T65.92*; T71.1{1-3,5,6,9}2*; T71.2{2,3}2* |
