# Supplementary material for: Using Machine Learning Imputed Outcomes to Assess Drug-Dependent Risk of Self-Harm in Patients with Bipolar Disorder: A Comparative Effectiveness Study
Source: JMIR Ment Health. 2021 Apr 21;8(4):e24522. doi: 10.2196/24522 (PMC8100888; doi:10.2196/24522)
Supplement: Multimedia Appendix 2 [file mental_v8i4e24522_app2.docx]

**Multimedia Appendix 2. Drugs of interest, analyzed either individually or as part of a composite class.** Only drugs/drug classes with ≥1000 prescription fills and ≥5 imputed and/or coded self-harm outcomes were selected eventually for the final regression analysis.

| **Treatment** | **RxNorm** | **Treatment** | **RxNorm** |
| --- | --- | --- | --- |
| **First generation antipsychotics (FGAs)** | | **Antidepressants** | |
| chlorpromazine | 2403 | **Monoaminooxidase inhibitors (MAOIs)** | |
| fluphenazine | 4496 | isocarboxazid | 6011 |
| haloperidol | 5093 | phenelzine | 8123 |
| loxapine | 6475 | selegiline | 9639 |
| perphenazine | 8076 | tranylcypromine | 10734 |
| pimozide | 8331 | **Noradrenergic and specific serotonergic antidepressants (NASSA)** | |
| promazine | 8742 | mirtazapine | 15996 |
| thioridazine | 10502 | **Norepinephrine–dopamine reuptake inhibitors (NDRIs)** | |
| thiothixene | 10510 | bupropion | 42347 |
| trifluoperazine | 10800 | **Serotonin–norepinephrine reuptake inhibitors (SNRIs)** | |
| triflupromazine | 10805 | desvenlafaxine | 734064 |
| **Lithium (as its own class)** | | duloxetine | 72625 |
| lithium carbonate | 42351 | levomilnacipran | 1433212 |
| **Mood stabilizing anticonvulsants (MSAs)** | | milnacipran | 588250 |
| carbamazepine | 2002 | venlafaxine | 39786 |
| lamotrigine | 28439 | **Selective serotonin reuptake inhibitors (SSRIs)** | |
| oxcarbazepine | 32624 | citalopram | 2556 |
| valproate | 40254 | escitalopram | 321988 |
| **Second generation antipsychotics (SGAs)** | | fluoxetine | 4493 |
| asenapine | 784649 | fluvoxamine | 42355 |
| clozapine | 2626 | paroxetine | 32937 |
| iloperidone | 73178 | sertraline | 36437 |
| lurasidone | 1040028 | vilazodone | 1086769 |
| olanzapine | 61381 | vortioxetine | 1455099 |
| paliperidone | 679314 | **Tri-and tetracyclic antidepressants** | |
| quetiapine | 51272 | amoxapine | 722 |
| risperidone | 35636 | clomipramine | 2597 |
| ziprasidone | 115698 | desipramine | 3247 |
| **Third generation antipsychotics (TGAs)** | | doxepin | 3638 |
| aripiprazole | 89013 | imipramine | 5691 |
| brexpiprazole | 1658314 | maprotiline | 6646 |
|  |  | protriptyline | 8886 |
|  |  | trimipramine | 10834 |
