# Supplementary figures and images for: Using Machine Learning Imputed Outcomes to Assess Drug-Dependent Risk of Self-Harm in Patients with Bipolar Disorder: A Comparative Effectiveness Study
Source: JMIR Ment Health. 2021 Apr 21;8(4):e24522. doi: 10.2196/24522 (PMC8100888; doi:10.2196/24522)

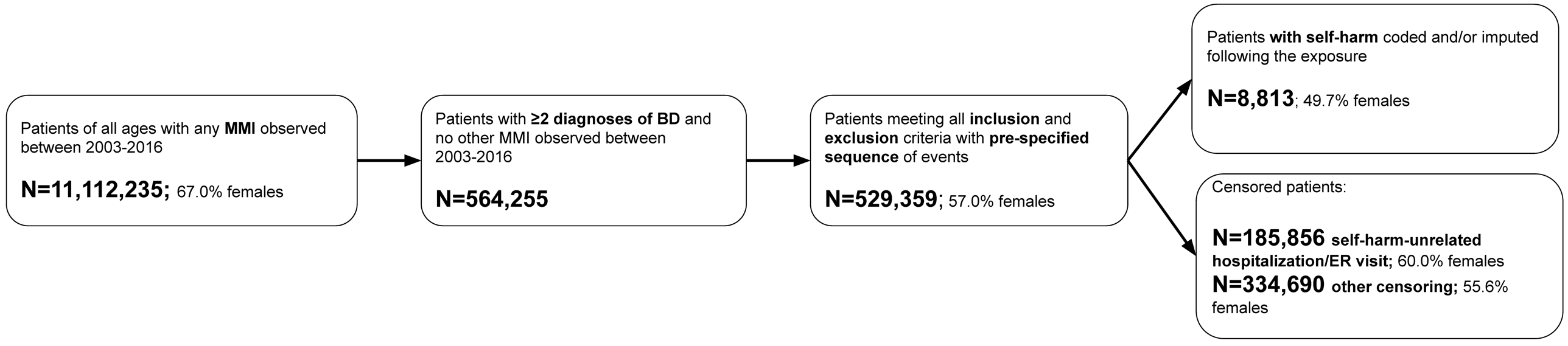

Supplement: Multimedia Appendix 3 [file mental_v8i4e24522_app3.png]

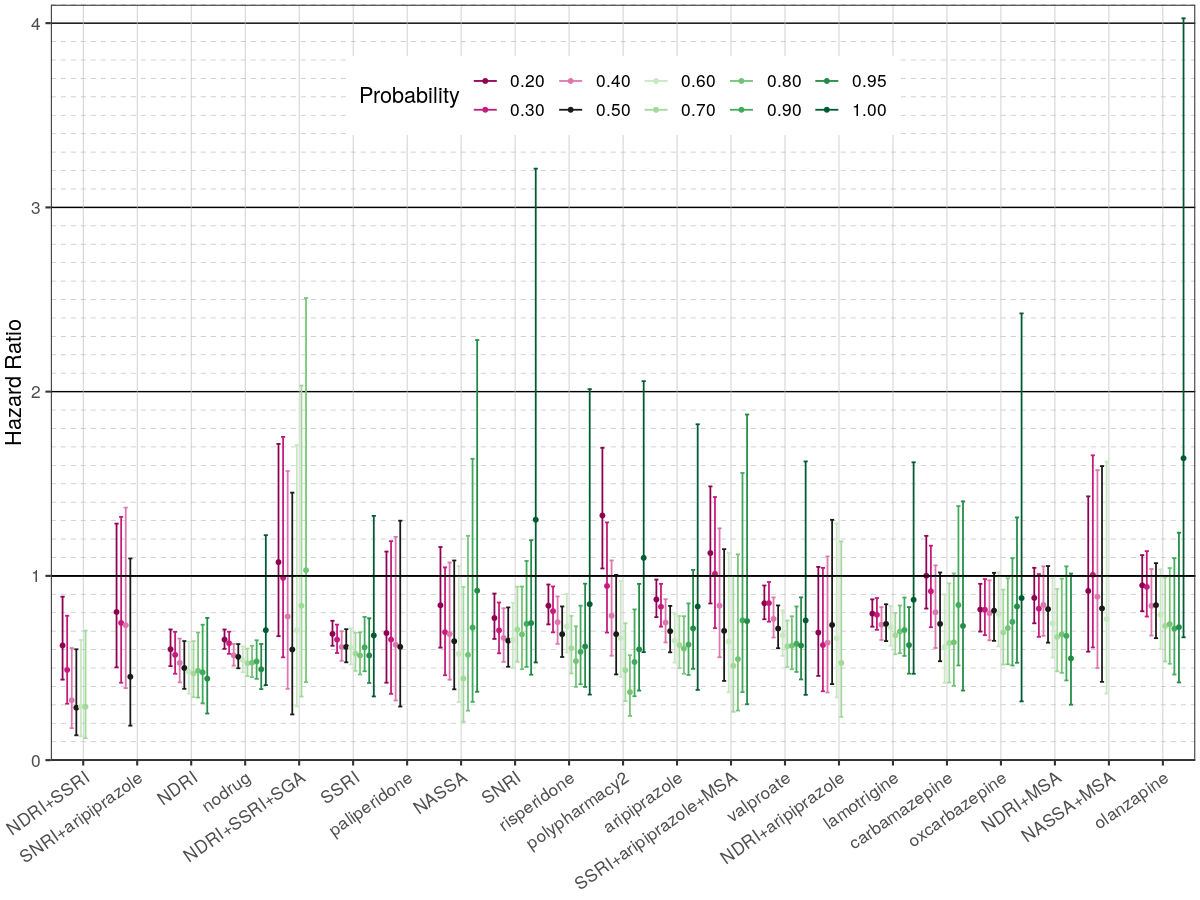

Supplement: Multimedia Appendix 4 [file mental_v8i4e24522_app4.png]

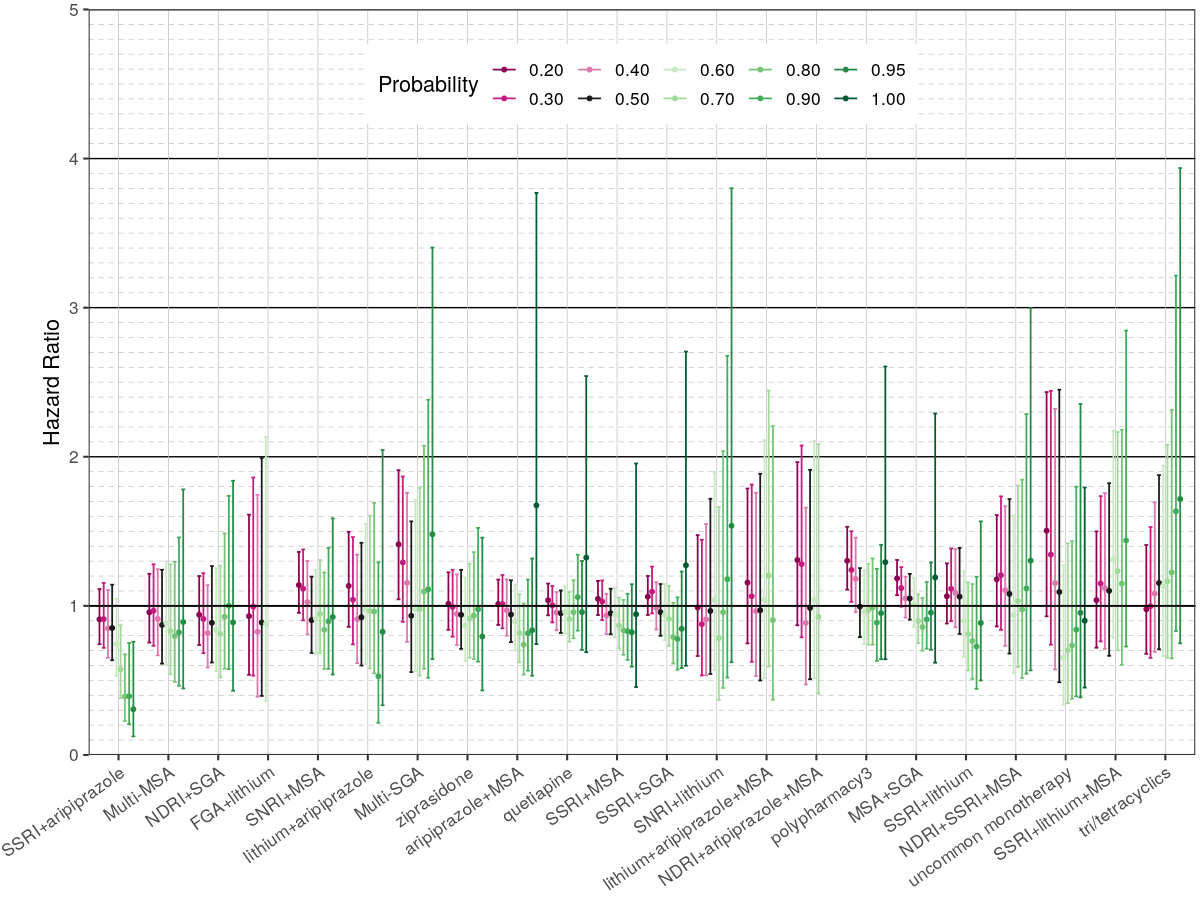

Supplement: Multimedia Appendix 5 [file mental_v8i4e24522_app5.png]

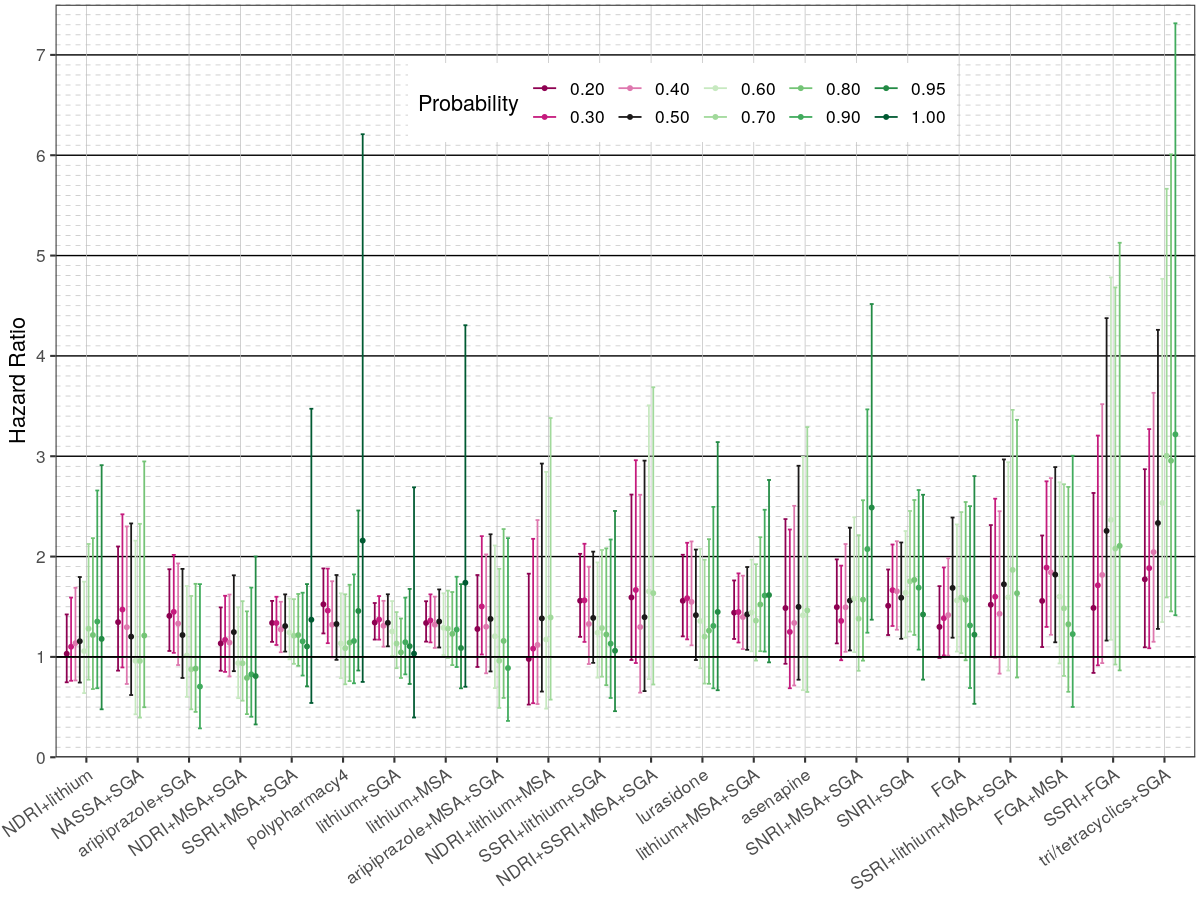

Supplement: Multimedia Appendix 6 [file mental_v8i4e24522_app6.png]

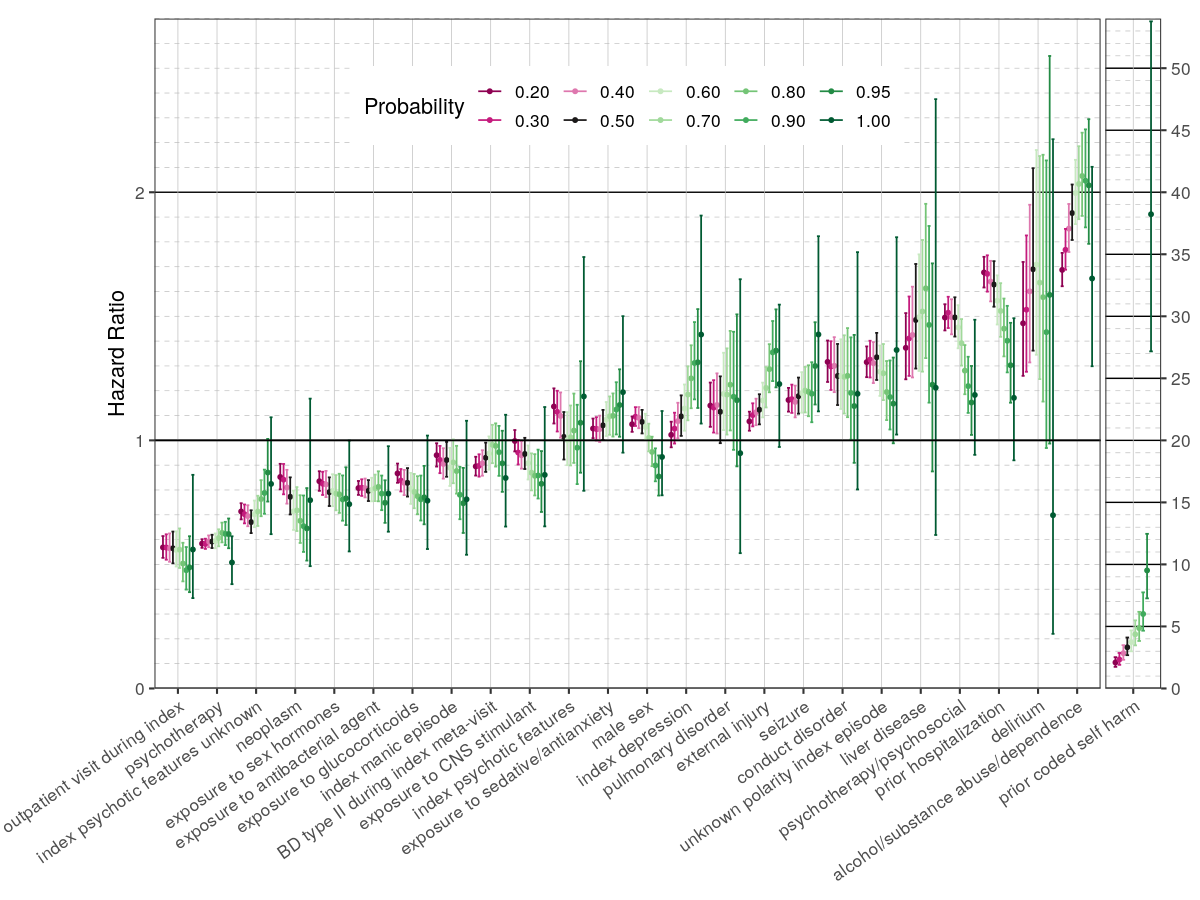

Supplement: Multimedia Appendix 7 [file mental_v8i4e24522_app7.png]
